# Transcriptome Analysis Reveals Potential Roles of Abscisic Acid and Polyphenols in Adaptation of *Onobrychis viciifolia* to Extreme Environmental Conditions in the Qinghai-Tibetan Plateau

**DOI:** 10.3390/biom10060967

**Published:** 2020-06-26

**Authors:** Hengxia Yin, Huakun Zhou, Wenying Wang, Lam-Son Phan Tran, Benyin Zhang

**Affiliations:** 1 State Key Laboratory of Plateau Ecology and Agriculture, Qinghai University, Xining 810016, China; hengxiayin@qhu.edu.cn; 2 The Key Laboratory of Restoration Ecology in Cold Region of Qinghai Province, Northwest Institute of Plateau Biology, Chinese Academy of Science, Xining 810008, China; hkzhou@nwipb.cas.cn; 3 School of Life Science, Qinghai Normal University, Xining 810008, China; wenyingwang@qhnu.edu.cn; 4 Institute of Research and Development, Duy Tan University, 03 Quang Trung, Da Nang 550000, Vietnam; 5 College of Eco-Environmental Engineering, Qinghai University, Xining 810016, China

**Keywords:** *Onobrychis viciifolia*, RNA-sequencing, extreme natural environment, phytohormone pathways, biosynthesis of polyphenols, abscisic acid, Qinghai-Tibetan Plateau

## Abstract

A detailed understanding of the molecular mechanisms of plant stress resistance in the face of ever-changing environmental stimuli will be helpful for promoting the growth and production of crop and forage plants. Investigations of plant responses to various single abiotic or biotic factors, or combined stresses, have been extensively reported. However, the molecular mechanisms of plants in responses to environmental stresses under natural conditions are not clearly understood. In this study, we carried out a transcriptome analysis using RNA-sequencing to decipher the underlying molecular mechanisms of *Onobrychis viciifolia* responding and adapting to the extreme natural environment in the Qinghai-Tibetan Plateau (QTP). The transcriptome data of plant samples collected from two different altitudes revealed a total of 8212 differentially expressed genes (DEGs), including 5387 up-regulated and 2825 down-regulated genes. Detailed analysis of the identified DEGs uncovered that up-regulation of genes potentially leading to changes in hormone homeostasis and signaling, particularly abscisic acid-related ones, and enhanced biosynthesis of polyphenols play vital roles in the adaptive processes of *O. viciifolia*. Interestingly, several DEGs encoding uridine diphosphate glycosyltransferases, which putatively regulate phytohormone homeostasis to resist environmental stresses, were also discovered. Furthermore, numerous DEGs encoding transcriptional factors, such as members of the *myeloblastosis* (*MYB*), *homeodomain-leucine zipper* (*HD-ZIP*), *WRKY*, and *nam-ataf1,2-cuc2* (*NAC*) families, might be involved in the adaptive responses of *O. viciifolia* to the extreme natural environmental conditions. The DEGs identified in this study represent candidate targets for improving environmental stress resistance of *O. viciifolia* grown in higher altitudes of the QTP, and can provide deep insights into the molecular mechanisms underlying the responses of this plant species to the extreme natural environmental conditions of the QTP.

## 1. Introduction

As sessile organisms, plants are continuously exposed to a plethora of environmental stresses, including abiotic and biotic stresses, which severely constrain their growth and productivity. The major abiotic stresses are salinity, drought, heat, cold, chilling, flooding, ultraviolet (UV) radiation, and heavy metal toxicity [1,2,3,4,5]. Abiotic stress is the prominent threat in natural conditions under continuous climate change, and has caused more than 50% of crop losses worldwide [6]. A better understanding of plant resistance to abiotic stresses, especially under natural conditions, is therefore of fundamental importance and has been one of the major research topics studied for improving plant production.

Facing with various environmental threats, plants have evolved a diverse array of protective mechanisms at biochemical, molecular, and physiological levels, with responses and changes in hormone homeostasis, transcriptional factors (TFs), photosynthesis, antioxidants, and the biosyntheses of osmotic adjustment-related substances [7,8,9,10,11]. The responsive mechanisms of plants to various specific abiotic/biotic stress conditions have been extensively investigated in the past decades; however, many studies have mainly focused on a single stress factor. Consequently, these findings could not provide a whole picture on how a given plant species may respond to combined stresses under truly uncontrolled natural conditions [12]. For example, the model plant *Arabidopsis thaliana* presented a specific molecular responsive mechanism in which over 770 specific transcripts were only displayed under combined drought and heat stress in comparison to the single stress [7]. Similar changes in metabolite and protein contents were also found, in which several metabolites and at least 45 proteins were only accumulated under the stress combination of drought and heat [7,8]. Additionally, transcriptome analyses of the sunflower (*Helianthus annuus*) plants exposed to a combination of heat and intensive light stress revealed numerous specific transcripts, compared with individual stresses [13]. These findings together indicated that the responsive mechanisms of plants to existing stresses under natural conditions are quite complicated. Unlike single or combined stresses, plants under natural conditions will be exposed to cross interactions of multiple stress factors, the consequence of which will not just be overlaid with their separately triggering responses. However, responsive mechanisms of plants to natural conditions, especially the extreme environment, still remain elusive. To further complicate the situation, the responses may also vary dependently on plant species.

In the past 10 to 15 years, the application of transcriptomics has substantially facilitated a deep understanding of responsiveness or resistance mechanisms of plants in the face of various specific stresses at a molecular level, especially with regard to unravelling differentially expressed genes (DEGs), signaling pathways and regulation of metabolic networks involved in responses to diverse stresses [14,15]. For example, an increasing number of TFs involved in plant responses to abiotic stresses have been discovered in many plant species, such as C-repeat binding factor/dehydration responsive element-binding (CBF/DREB), myeloblastosis (MYB), WRKY, nam-ataf1,2-cuc2 (NAC) and basic region/leucine zipper motif (bZIP) TFs, the biological functions of which have been confirmed by transgenic studies [16,17,18,19,20]. Additionally, molecular mechanisms underlying the metabolic changes and signal transduction in plant responses to stresses have been extensively documented [16,17,18,19,20,21]. Phytohormone pathways, including those of abscisic acid (ABA), ethylene, cytokinin (CK), indole 3-acetic acid (IAA), gibberellic acid (GA), and jasmonic acid (JA), play crucial roles in the regulation of plant resistance to various abiotic or biotic stresses [20,21,22]. For instance, the biosynthesis of ABA is known to be promoted under drought and osmotic stress, which triggers significant transcriptional, biochemical, and physiological changes in stressed plants [23,24,25].

*Onobrychis viciifolia*, also called sainfoin, belonging to Leguminosae, is a perennial forage crop widely planted in the Qinghai-Tibetan Plateau (QTP). *O. viciifolia* contains condensed tannins (CTs), or proanthocyanidins that belong to a class of polyphenols [26]. CTs in high concentrations are thought to contribute to reduce methane emissions, and increase nitrogen-use efficiency, anthelmintic properties, as well as bloat prevention for ruminants in contrary to other legumes like alfalfa (*Medicago sativa*) [27,28]. Given the importance of CTs for considering *O. viciifolia* as an excellent forge crop, numerous studies have focused on their composition and identification in recent years [26,29,30,31,32]. In addition to CTs, a range of other types of chemical compounds, such as flavonoids, isoflavonoids, and hydroxycinnamic acids, are also enriched in *O. viciifolia*, and were proposed to be correlated with its drought tolerance [33]. Furthermore, *O. viciifolia* has better forage quality and higher total sugar and crude protein contents than the other forage legumes like trefoil (*Lotus corniculatus*) and chicory (*Cichorium intybus*) [34]. Therefore, *O. viciifolia* wins a reputation as ‘the queen of forages’ due to these benefits that suggest *O. viciifolia* to be an alternative to *M. sativa* as a valuable forage crop in the QTP [35].

Being known as the Earth’s third pole and the highest plateau (average elevation of more than 4000 m above sea level), the QTP possesses extreme environmental factors, including low temperature, strong UV radiation, severe drought, low oxygen, and soil nutritional deficiency [36,37,38]. Notably, *O. viciifolia* appears to be increasingly resistant to extreme natural conditions of higher altitudes in the QTP, showing better overwintering and higher yields year after year. Thus, *O. viciifolia* is recognized as an ideal plant material for investigating the molecular mechanisms of plants in acclimatization to extreme circumstances in the QTP. Such mechanisms of *O. viciifolia* with regard to the QTP natural conditions have not been reported yet. Thus, to obtain a global molecular understanding, and identify key responsive genes of the forage crop *O. viciifolia*, which are involved in its acclimatization to extreme natural conditions, we applied RNA-sequencing (RNA-seq) to obtain transcriptomes of *O. viciifolia* plants naturally planted at Tongde (TD, high altitude) and Minhe (MH, low altitude) counties in the QTP for a comparative transcriptome analysis.

Based on the climatic parameters from Qinghai Institute of Meteorology Science, these two counties have significantly different climatic characteristics due to location at different altitudes. In recent three years, TD has the annual average temperature of 3.92 °C, annual average precipitation of 611.4 mm, and annual accrued sunlight of 2476.4 h, whereas MH has relative mild climatic factors with the annual average temperature of 9.44 °C, annual average precipitation of 407.7 mm, and annual accrued sunlight of 2335.5 h. Although the annual average precipitation in TD is higher than that in MH, the annual average water evaporation in TD (~1616.7 mm) is almost 3-times higher than that at MH (~629.7 mm), resulting in a more water-deficit TD when compared with MH. Thus, comparative transcriptome analysis of *O. viciifolia* plants naturally planted at the high-altitude TD and low-altitude MH with different natural conditions will allow us to understand the acclimatization process of *O. viciifolia* to extreme natural conditions occurring at higher altitude. Additionally, this work will also provide the first-ever transcriptome resource for *O. viciifolia* field-grown in the two different altitudes of the QTP. Identification of DEGs from this study will therefore help decipher the molecular mechanisms underlying the responses of this forage crop to their surrounding detrimental natural conditions, which could contribute to genetic engineering and to improving the environmental stress resistance of *O. viciifolia* grown in higher altitudes of the QTP.

## 2. Materials and Methods

### 2.1. Plant Materials

*Onobrychis viciifolia* was planted in May at the germplasm breeding farms of Tongde (TD, E 100°39′26′′, N 35°09′45′′; H 3830 m) and Minhe (MH, E 102°34′46′′, N 36°15′56′′; H 1840 m) counties that are located in the three-river headwater region and the most eastern edge of the QTP in Qinghai province, respectively. To elucidate the stress resistance mechanisms under natural plateau conditions, two-year-old *O. viciifolia* forages field-grown at TD and MH (around 80–100 cm in height) were collected in the seventh day during flowering on 6 July and 23 June in 2018, respectively, as investigated objectives. To avoid the sampling bias, the sampling time of the two locations were both at 10:00 a.m. with the same sampling method. Five plants were randomly selected for each repetition (5 independent plants/replication). Then, the three fresh and undamaged leaves from the middle position of the herbage (3 leaves from each plant) were collected and washed with distilled water, cut into pieces, and mixed together as a biological replicate. All collected samples were immediately frozen in liquid nitrogen and stored at −80 °C until further use. RNA-seq and RT-qPCR analyses were both performed using three independent biological replications.

### 2.2. Total RNA Extraction, and Construction and Sequencing of mRNA Libraries

Total RNA was extracted from the biological replicates using the MiniBEST kit (Takara, Dalian, China) following the manufacturer’s instructions, and the concentration and integrity of each RNA sample were determined using a NanoDrop (Thermo Scientific, Waltham, MA, USA) and an Agilent 2100 BioAnalyzer (Agilent Technologies, Palo Alto, CA, USA), respectively. Construction and sequencing of the cDNA libraries of six samples were performed at the Gene Denovo Biotechnology Co., Ltd., Guangzhou, China. All transcriptome sequencing data have been uploaded to the NCBI Sequence Read Archive (SRA) database (https://www.ncbi.nlm.nih.gov/sra) under the SRA accession number PRJNA612135.

### 2.3. Transcriptome Sequencing Data Processing and Analysis

The clean reads, namely high-quality reads, were obtained by removing the adapters, ploy-N sequences, and low-quality (Q-value ≤ 10) reads from the raw reads. Meanwhile, the values of Q20, Q30, N50, and GC content of the clean reads were calculated. Transcriptome de novo assembly was then carried out with the clean reads using the Trinity Program with default parameters [39]. Clean datasets of six libraries were pooled for de novo assembly to create a reference genome of *O. viciifolia*. The annotation of assembled unigenes was conducted using the nucleotide Basic Local Alignment Search Tool (BLASTn) against various public databases, including the Nr (https://ftp.ncbi.nlm.nih.gov/blast/db/FASTA/), Swiss-Prot (https://www.uniprot.org/), KOG (https://ftp.ncbi.nih.gov/pub/COG/KOG/) and KEGG (KEGG, https://www.kegg.jp/) ortholog databases, with an e-value cutoff of 10^−10^. Gene expression levels were calculated and normalized to reads per kilobases per million reads (RPKM) values [40].

### 2.4. Identification and Functional Annotation of DEGs

DEGs across the samples were identified according to their expression levels with the thresholds of |log_2_ (fold change)| ≥  1 and *p*-value < 0.05. The GO and KEGG enrichment analyses for *O. viciifolia* were conducted using the GO and KEGG databases to gain functional annotation of the DEGs. TFs were predicted by searching against the Plant Transcriptional Factor Database (PlantTFDB 4.0, http://www.plntfdb.bio.uni-potsdam.de/v3.0/) [41]. Further annotation and classification of DEGs were visualized in the MapMan version 3.6.0RC1, and the annotated files for MapMan visualization were generated with Mercator version 3.6 (https://www.plabipd.de/portal/web/guest/mercator-sequence-annotation) [42].

### 2.5. Phylogenetic Analysis

Phylogenetic tree was created using internal transcribed spacer sequences of indicated legume species and *A. thaliana*, which were downloaded from Genebank with the following accession numbers: AF053171.1 (*T. pretense*), JQ858260.1 (*M. truncatula*), AY594660.1 (*C. arietinum*), JQ858258.1 (*Lotus japonicus*), EU727147.1 (*Vigna angularis*), MH547408.1 (*Phaseolus vulgaris*), MG991099.1 (*C. cajan*) as well as AJ232900.1 (*A. thaliana*) that was set as an outgroup. The phylogenetic tress was constructed by the Neighbor-Joining method using software MEGA 6.0 with 1000 bootstrap.

### 2.6. RT-qPCR

Genomic DNA was removed from extracted total RNA using the PrimeScriptRT reagent Kit with gDNA Eraser (Takara, Dalian, China). DNA-free total RNA samples were then used to synthesize cDNA using the PrimeScriptRT reagent kit with the gDNA Eraser (Takara, Dalian, China), according to the manufacturer’s instructions. Detailed information on the primers used in the study is provided in Supplementary Table S10. The transcriptional levels of selected DEGs were detected using the TB Green Premix Ex Taq^TM^ II (Tli RNaseH Plus) (Takara, Dalian, China) and the LightCycler 480 System (Roche, Basel, Switzerland). Three independent biological replicates were used in the RT-qPCR analysis, and the relative fold change was calculated based on the Ct values, with *18S rRNA* being used as a reference gene [43].

### 2.7. Determination of the Contents of Total Flavonoids, Total Anthocyanins and Other Flavonoid Compounds

Fresh leaves of *O. viciifolia* plants were dried with a vacuum freeze dryer (EYELA, Tokyo, Japan), and then ground into powder and subjected to a sieve with aperture size of 0.15 mm. Subsequently, 0.1 g sample was then extracted with 1 mL methanol for 2 h at 60 °C using the ultrasonic extraction method, then centrifuged for 15 min at a speed of 13,500 rpm. The supernatant was subsequently used for determination of the contents of total flavonoids and total anthocyanins using the aluminum colorimetric method as described previously at the wavelengths of 510 and 530 nm, respectively, with rutin as a standard [44,45].

The qualitative and quantitative analyses of four flavonoid compounds rutin, quercetin, kaempferol, and myricetin were performed using the LC-20AT equipment equipped with a PDA detector (Shimadzu, Japan). Standard solutions of rutin, quercetin, kaempferol, and myricetin (purchased from Chengdu Herbpurity Co. Ltd., Chengdu, China) were dissolved in methanol at a final concentration of 0.37–0.60 mg/mL. The injection volume of samples or standards was 10 uL in each experiment. HPLC analysis of flavonoids was performed on a reverse phase C18 column (250 mm × 4.6 mm, 5 μm, Shimadzu, Japan) at a flow rate of 1 mL/min over a 60 min gradient program with 0.5% formic acid (solvent A) and acetonitrile containing 0.1% formic acid (solvent B) in a linear step: T = 0 min, 5% solvent B; T = 30 min, 35% solvent B; T = 45 min, 70% solvent B; T = 52 min, 100% solvent B; B; T = 53 min, 5% solvent B; T= 60 min, 5% solvent B. The column temperature was set at 30 °C.

## 3. Results

### 3.1. Transcriptome Profiling of O. viciifolia Grown under Two Different Natural Environmental Conditions

To understand how *O. viciifolia* plants acclimatize to extreme natural conditions under high altitude in the QTP at molecular level, we first obtained two comprehensive transcriptome profiles of leaves of *O. viciifolia* grown at two different altitudes of the QTP. Totally, six RNA-seq libraries of leaves collected from *O. viciifolia* plants planted in the high-altitude TD (E 100°39′26′’, N 35°09′45′’; H 3830 m) and low-altitude MH (E 102°34′46′’, N 36°15′56′’; H 1840 m) regions were generated. After conducting RNA-seq with the Illumina HiSeq 4000 platform, a total of 44,392,764, 81,922,056, 69,023,986, 53,001,304, 44,778,542, and 48,966,846 raw reads were obtained from three high-altitude plant samples (TD group; TD-1, TD-2, and TD-3) and three low-altitude plant samples (MH group; MH-1, MH-2, and MH-3), respectively (Table 1). Subsequently, low-quality reads and adapters were stringently trimmed, and a total of ~556 million high-quality reads accounting for more than 97.60% of the raw reads were obtained. De novo transcriptome assembly using Trinity [39] generated 74,791 unigenes, with an average sequence length of 820 bp and an average N50 of 1458 bp. The lengths of these unigenes were mainly distributed in the range of 200–500 bp (40,604 unigenes), followed by 500–1000 bp (14,805 unigenes), 1000–2000 bp (12,038 unigenes), and >2000 bp (7344 unigenes) (Supplementary Figure S1).

### 3.2. Annotation of Assembled Unigenes

To obtain the maximum number of annotated genes, all of the assembled unigenes were searched against public databases, namely the Non-Redundant Protein Sequence Database (Nr), Swiss Institute of Bioinformatics databases (Swiss-Prot), Kyoto Encyclopedia of Genes and Genomes (KEGG), and EuKaryotic Orthologous Groups (KOG) databases. In total, 46,508 unigenes were annotated by four databases, accounting for 62.18% of all assembled unigenes. Specifically, the numbers of unigenes with significant similarity to sequences annotated by Nr, Swiss-Prot, KOG and KEGG databases were 43,717 (58.45%), 32,973 (44.09%), 24,841 (33.21%), and 16,305 (21.08%), respectively, with an e-value ≤ 10^−5^ (Table 2 and Supplementary Figure S2).

**Figure 1:**
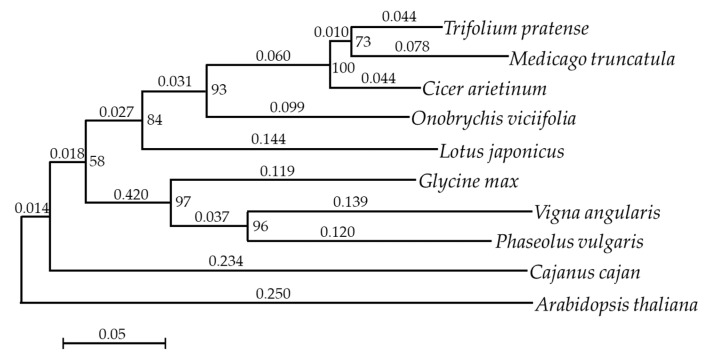
Phylogenetic relationships of *Onobrychis viciifolia* with its related legumes. *Arabidopsis thaliana* was included as an outgroup. Numbers at the branchpoints and above the branches represent the percentage of bootstrap values and distances between clusters, respectively.

A further analysis of annotated sequences revealed the best hits to *Medicago truncatula* (24.92% matched genes), followed by *Cicer arietinum* (20.98% matches), *Glycine max* (7.64%), and *Cajanus cajan* (7.22%) (Table 3). This result was reliable and could be explained by the fact that *O. viciifolia* is the closest species to *M. truncatula* and *C. arietinum* in terms of the phylogenetic relationship [46,47]. Additionally, a phylogenetic tree of *O. viciifolia* and its related legumes was generated, which also demonstrated the closest relationships of *O. viciifolia* with *C. arietinum* and *M. truncatula* (Figure 1).

### 3.3. DEGs from O. viciifolia Plants Grown at Different Altitudes

Since we were interested in exploring the molecular changes of *O. viciifolia* plants in acclimatization to high-altitude natural conditions, the samples harvested at low altitude were used as the control in the comparison. Thus, leaf transcriptome data of *O. viciifolia* plants grown at high-altitude TD and low-altitude MH were used in subsequent comparative transcriptome analyses (TD/MH comparison hereafter). A total of 8212 differentially expressed genes (DEGs) were identified in TD/MH with the threshold of |log_2_ (fold change, FC)| ≥  1 and *p* < 0.05. Of these DEGs, 5387 unigenes were up-regulated and 2825 unigenes were down-regulated in TD/MH comparison (Supplementary Table S1). The top 50 DEGs are displayed in Supplementary Table S2.

**Figure 2:**
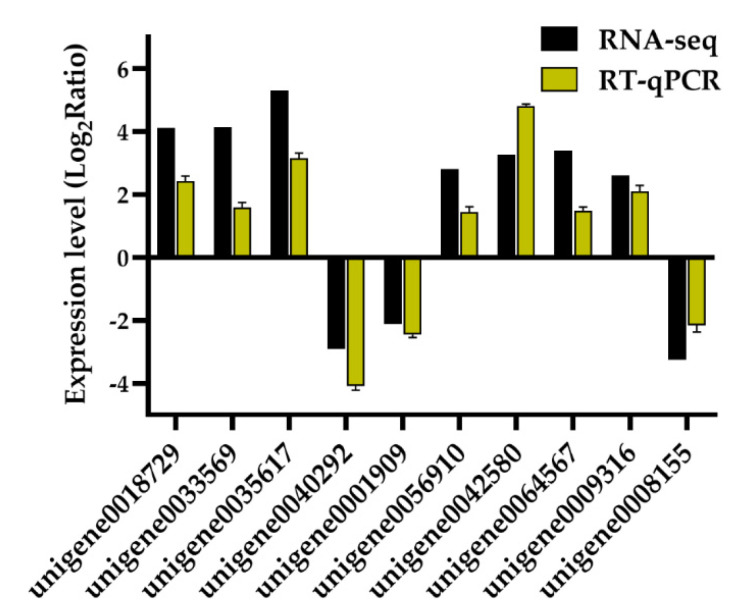
Validation of 10 differentially expressed genes (DEGs) obtained from the comparison of leaf transcriptomes of *Onobrychis viciifolia* plants grown at high-altitude Tongde (TD) and low-altitude Minhe (MH) using RT-qPCR. The Y-axis represents expression changes in log_2_ (fold change) of ten DEGs (TD/MH comparison) obtained by RNA-sequencing (RNA-seq) and RT-qPCR methods. The X-axis indicates randomly selected DEGs for RT-qPCR validation. Bars represent means ± SDs of three biological replicates.

Ten DEGs were randomly chosen to verify the reliability of the obtained RNA-seq data using real time-quantitative PCR (RT-qPCR). The results showed that the tendency of transcriptional changes of all tested genes were consistent between the two methods (Figure 2 and Supplementary Figure S3), confirming the accuracy of our high-throughput RNA-seq data.

### 3.4. Functional Enrichment Analysis of DEGs

**Figure 3:**
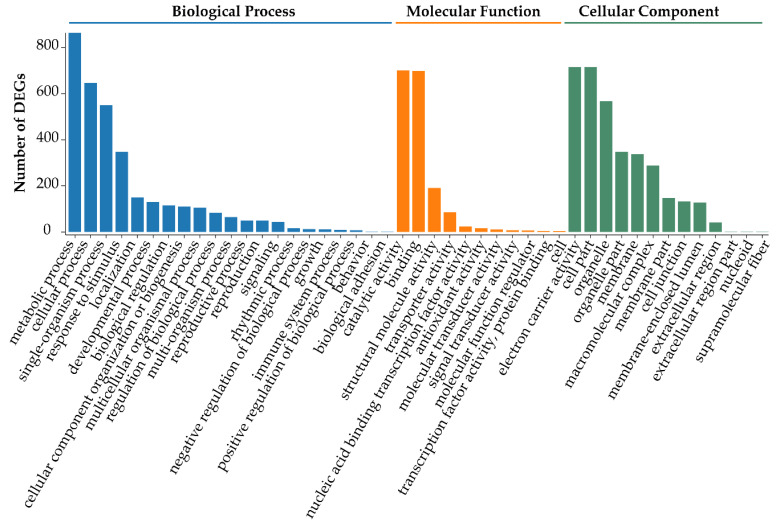
Functional classification of the differentially expressed genes (DEGs) obtained from the comparison of leaf transcriptomes of *Onobrychis viciifolia* plants grown at high-altitude Tongde (TD) and low-altitude Minhe (MH). Unigenes with BLAST hits were classified into three major categories, namely ‘Biological Process’, ‘Molecular Function’, and ‘Cellular Component’, according to a GO analysis. The Y-axis represents the number of DEGs (TD/MH comparison) in each subcategory. The X-axis indicates the most enriched 45 GO subcategories.

To further understand the functions of DEGs obtained from TD/MH comparison, gene ontology (GO, http://geneontology.org/) enrichment analysis was carried out to functionally classify the DEGs. Overall, the identified DEGs were enriched in 45 GO terms and assigned to the three categories of the GO database, namely ‘Biological Process’ (21 subcategories), ‘Cellular Component’ (13 subcategories), and ‘Molecular Function’ (11 subcategories) (Figure 3). GO subcategories ‘metabolic process’, ‘cellular process’, and ‘single-organism process’ in ‘Biological Process’, ‘catalytic activity’ and ‘binding’ in ‘Molecular Function’, and ‘electron carrier activity’, ‘cell part’, and ‘organelle’ in ‘Cellular Component’ were significantly enriched (Figure 3 and Supplementary Table S3).

In addition, the GO terms ‘response to light intensity’ (GO:0009642), ‘response to UV’ (GO:0009411), ‘rhythmic process’ (GO:0048511), and ‘response to osmotic stress’ (GO:0006970) were highly enriched with the DEGs (Supplementary Table S3), further confirming that the influence of solar radiation on gene expression patterns in plants at the higher altitude was more remarkable than that at the lower altitude [48]. Furthermore, of the 50 DEGs shown in Supplementary Table S2, numerous genes were involved in the biological processes responding to environmental stimuli, such as unigene0043850 (log_2_ (FC) = 4.34), unigene0054807 (log_2_ (FC) = 5.54), and unigene0060124 (log_2_ (FC) = 3.77). Interestingly, the highly expressed unigene0054137 (log_2_ (FC) = 11.43) was annotated to participate in the biological processes of ‘response to radiation’, ‘response to abiotic stimulus’, ‘rhythmic process’, and ‘long-day photoperiodism, flowering’ (Supplementary Table S3), suggesting that it might be involved in responses to solar radiation, light intensity and photoperiod. Thus, these results collectively indicate that the specific QTP natural conditions at higher altitude, such as strong solar radiation and long-day periods in the summer, will inevitably induce gene expression changes in *O. viciifolia*, leading to its acclimatization responses to such conditions. The unigene0054137, therefore, might be a promising candidate target for improving resistance of *O. viciifolia* to natural conditions of higher altitude.

**Figure 4:**
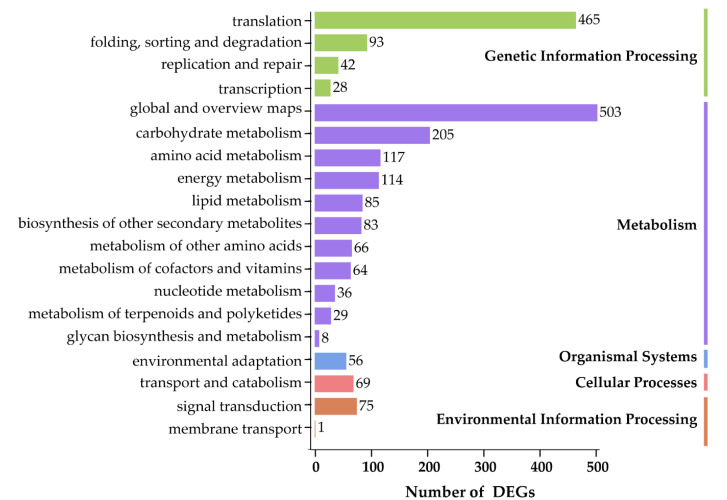
KEGG enrichment analysis of differentially expressed genes (DEGs) in leaves of *Onobrychis viciifolia* plants collected from the high-altitude Tongde (TD) versus the low-altitude Minhe (MH). The Y-axis represents the KEGG terms. The X-axis represents the number of DEGs (TD/MH comparison). The number corresponding to each KEGG term indicates the number of enriched DEGs in each term. All KEGG pathways are classified into five categories, namely ‘Genetic Information Processing’, ‘Metabolism’, ‘Organismal Systems’, ‘Cellular Processes’, and ‘Environmental Information Processing’ as displayed by different colors.

To dissect the metabolic pathways, in which the DEGs are involved, the identified DEGs were mapped to the KEGG database. In total, 19 KEGG pathways were enriched into five categories, including ‘Genetic Information Processing’ (4 terms), ‘Metabolism’ (11 terms), ‘Organism Systems’ (1 term), ‘Cellular Processes’ (1 term), and ‘Environmental Information Processing’ (2 terms), suggesting that the most enriched metabolic processes could predominantly act in responses to environmental cues (Figure 4). Interestingly, DEGs were mainly overrepresented in ‘global and overview maps’, followed by ‘translation’, ‘carbohydrate metabolism’, ‘amino acid metabolism’, and ‘energy metabolism’ KEGG terms (Figure 4).

**Figure 5:**
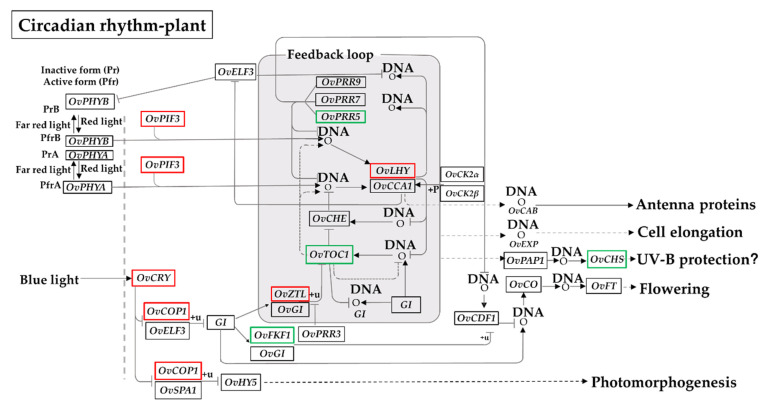
A putative ‘circadian rhythm–plant’ pathway was generated from the KEGG database based on the log_2_ (FC) values of identified differentially expressed genes (DEGs). The symbols in boxes indicate the enzyme codes. Red and green colors depict the up- and down-regulated genes, respectively. *OvPHYA*, *phytochrome A*; *OvPHYB*, *phytochrome B*; *OvELF3*, *EARLY FLOWERING 3*; *OvPIF3*, *phytochrome-interacting factor 3*; *OvPRR5*, *pseudo-response regulator 5*; *OvPRR7*, *pseudo-response regulator 7*; *OvPRR9*, *pseudo-response regulator 9*; *OvCRY*, *cryptochrome*; *OvCOP1*, *constitutive photomorphogenic 1*; *OvSPA1*, *suppressor of PHYA-105 1*; *OvGI*, *GIGANTEA*; *OvHY5*, *transcriptional factor HY5*; *OvCHE*, *transcription factor TCP21*; *OvZTL*, *ZEITLUPE*; *OvLHY*, *late elongated hypocotyl*; *OvCCA1*, *circadian clock associated 1*; *OvFKF1*, *flavin-binding kelch repeat F-box protein*; *OvCDF1*, *cycling dof factor 1*; *OvCHS*, *chalcone synthase*; *OvTOC1*, *timing of cab expression 1*; *OvCO*, *zinc finger protein CONSTANS*; *OvFT*, *FLOWERING LOCUS T*; *OvCK2α*, *casein kinase II subunit alpha*; *OvCK2β*, *casein kinase II subunit beta*.

Given the difference in daily cycles between the high and low altitudes [49], we next took a closer look at the putative functions of the DEGs involved in the ‘circadian rhythm–plant’ pathway using a KEGG analysis (Figure 5 and Supplementary Table S4). The DEGs encoding phytochrome-interacting factor 3 (*OvPIF3*; unigene0042002, (log_2_ (FC) = 1.51)), cryptochrome (*OvCRY*; unigene0013838, (log_2_ (FC) = 1.01); unigene0054923, (log_2_ (FC) = 1.85); unigene0065731, (log_2_ (FC) = 1.41)), constitutive photomorphogenic 1 (*OvCOP1*; unigene0001254, (log_2_ (FC) = 1.50)), ZEITLUPE (*OvZTL*; unigene0039545, (log_2_ (FC) = 1.19)), and late elongated hypocotyl (*OvLHY*; unigene0039545, (log_2_ (FC) = 1.19); unigene0067673, (log_2_ (FC) = 6.42)) were significantly up-regulated (Figure 5). In contrast, the down-regulated DEGs involved in the ‘circadian rhythm–plant’ pathway consisted of pseudo-response regulator 5 (*OvPRR5*; unigene0001332, (log_2_ (FC) = −1.37)), flavin-binding kelch repeat F-box protein 1 (*OvFKF1*; unigene0035551, (log_2_ (FC) = −2.09)), chalcone synthase (*OvCHS*; unigene0004754, (log_2_ (FC) = −1.70); unigene0016510, (log_2_ (FC) = −2.40)), and timing of cab expression 1 (*OvTOC1*; unigene0002982, (log_2_ (FC) = −2.12)). The positive roles of *PIF3*, *CRY,* and *PRR5* in responses to various abiotic stresses have been well-documented in plants [50,51,52]. Our analysis suggested that these changes in gene expression may be closely related to the acclimatization of *O. viciifolia* plants to the long-day photoperiod occurring at higher altitude in the QTP.

### 3.5. Global Environmental Stress Responses Revealed by MapMan Analysis

**Figure 6:**
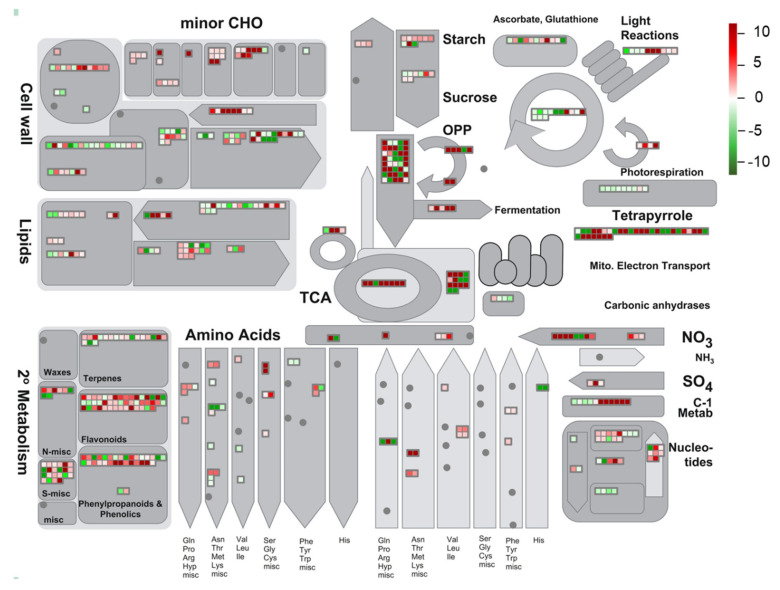
Global view of the identified differentially expressed genes (DEGs) involved in diverse metabolic pathways using a MapMan analysis. Gray shapes containing colored boxes represent different metabolic pathways with related DEGs. Heatmaps in gray boxes indicates expression changes of related genes in leaves of *Onobrychis viciifolia* plants collected from the high-altitude Tongde (TD) versus the low-altitude Minhe (MH). Red and green colors represent up-regulated and down-regulated DEGs (TD/MH comparison), respectively, based on their log_2_ (FC) values.

**Figure 7:**
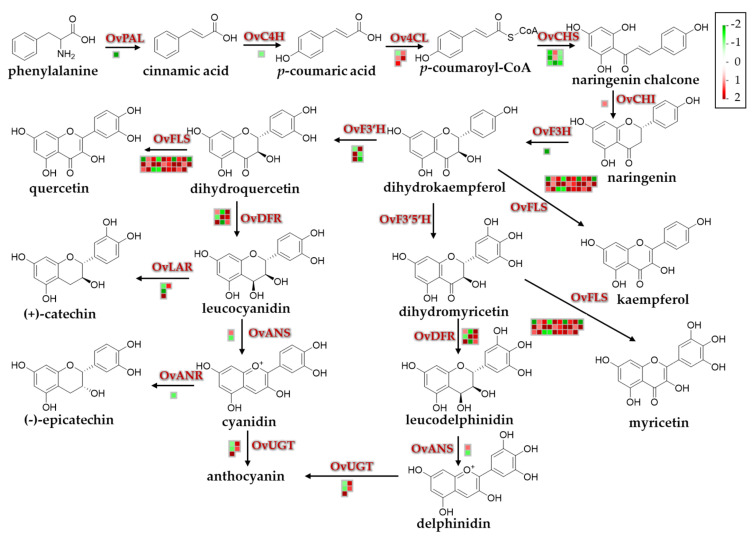
Flavonoid and anthocyanin biosynthetic pathways and expression changes of related genes in leaves of *Onobrychis viciifolia* grown at high-altitude Tongde (TD) and low-altitude Minhe (MH). Capital letters above/next to the arrows represent enzymes encoded by respective biosynthetic genes involved in every reaction step. The corresponding compound name is shown below each chemical structure. The details of key biosynthetic enzymes are as follows: OvPAL, phenylalanine ammonium lyase; OvC4H, cinnamic acid 4-hydroxylase; Ov4CL, 4-coumarate-CoA ligase; OvCHS, chalcone synthase; OvCHI, chalcone isomerase; OvF3H, flavanone-3-hydroxylase; OvF3′H, flavonoid-3′-hydroxylase; OvF3′,5′H, flavonoid-3′,5′-hydroxylase; OvFLS, flavonol synthase; OvDFR, dihydroflavonol reductase; OvANS, anthocyanin synthase; OvANR, anthocyanin reductase; OvUGT, uridine diphosphate glucosyltransferase. Colored boxes and scale indicate expression changes of related genes (TD/MH comparison). Red and green colors represent up- and down-regulation, respectively, based on log_2_ (FC) in expression values of related genes (TD/MH comparison).

To extend our understanding of the biological functions of the identified DEGs, a MapMan analysis was (http://mapman.gabipd.org/) performed to visualize the pathways involved in the environmental stress responses. The DEGs and their mapping files were annotated and generated with an online tool of the Mercator version 3.6 (https://www.plabipd.de/portal/web/guest/mercator-sequence-annotation) [42]. Results revealed that a total of 8212 DEGs were mapped to 845 pathways, of which 52 pathways were filtered and enriched by the DEGs with the cutoff *p* < 0.05 (Figure 6 and Supplementary Table S5). Among these metabolic pathways, the majority of DEGs were overrepresented in three pathways, namely ‘TCA/org. transformation. TCA’, ‘flavonoids’ and ‘phenylpropanoids and phenolics’ (Figure 6), suggesting that these pathways might contribute the most to resisting adverse natural conditions of the QTP. Due to the antioxidative functions of flavonoids and phenylpropanoids in many plant species under stress conditions [53,54], we more closely investigated the DEGs involved in their biosynthetic pathways (Figure 7 and Supplementary Figure S4; Supplementary Table S6). Genes encoding key enzymes involved in the flavonoid biosynthetic pathway, such as flavonol synthase (OvFLS; unigene0046947, log_2_ (FC) = 5.26), uridine diphosphate glycosyltransferase (OvUGT; unigene0015711, log_2_ (FC) = 1.92; unigene0046763, log_2_ (FC) = 1.96), ferulate 5-hydroxylase (OvF3′H; unigene0043777, log_2_ (FC) = 5.00), and dihydroflavonol reductase (OvDFR; unigene0002996, log_2_ (FC) = 9.78), were significantly up-regulated, whereas those encoding OvCHS (unigene0016510, log_2_ (FC) = −2.40; unigene0004754, log_2_ (FC) = −1.70) and flavanone-3-hydroxylase (OvF3H; unigene0051593, log_2_ (FC) = −2.20) were down-regulated (Figure 7). The number of genes putatively encoding OvFLS enzymes was mostly represented, suggesting that their end-products of three biosynthesized small molecules quercetin, kaempferol, and myricetin might play positive roles in promoting the environmental stress resistance of *O. viciifolia* grown in the higher altitudes of QTP (Figure 7). Furthermore, derivatives of these three compounds, such as glycosylated compounds [55], might also play substantial roles in the resistance of *O. viciifolia*.

In the phenylpropanoid biosynthetic pathway, genes encoding 4-coumarate-CoA ligase (*Ov4CL*; unigene0063881, log_2_ (FC) = 1.87; unigene0059064, log_2_ (FC) = 1.51), chalcone *O*-methyltransferase (OvChOMT; unigene0021581, log_2_ (FC) = 9.37; unigene0046268, log_2_ (FC) = 8.50), and cinnamoyl CoA reductase (OvCCR; unigene0039454, log_2_ (FC) = 3.10; unigene0059064, log_2_ (FC) = 1.51) showed distinct up-regulation, whereas those encoding hydroxycinnamoyl transferases (OvHCT; unigene0023010, log_2_ (FC) = −6.06; unigene0034833, log_2_ (FC) = −2.34) were down-regulated (Supplementary Figure S4 and Supplementary Table S6). The polyphenols like flavonoids and phenylpropanoids, as important components of the non-enzymatic antioxidant system have important roles in plant resistance to environmental stresses through scavenging excessive reactive oxygen species (ROS) induced by stresses [54]. The dramatic increase in biosynthesis of these secondary metabolites in the TD-grown plants, compared with the MH-grown plants, would aid *O. viciifolia* plants in their acclimatization to the extreme conditions in higher altitude regions of the QTP, such as extremely low temperature, drought, and strong UV radiation.

### 3.6. Responses of DEGs Involved in Phytohormone Pathways

**Figure 8:**
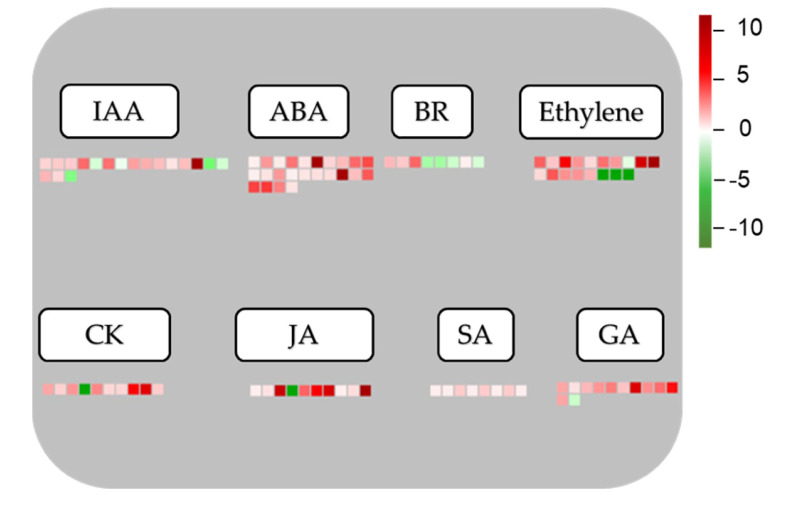
MapMan overview of differentially expressed genes (DEGs) related to phytohormone metabolic and signaling processes, which were identified in *Onobrychis viciifolia* plants of high (Tongde, TD) versus low (Minhe, MH) altitudes. Colored boxes and scale indicate expression changes of related genes. Red and green colors represent up- and down-regulation, respectively, based on log_2_ (FC) values of TD-grown versus MH-grown plants. IAA, indole 3-acetic acid; ABA, abscisic acid; BR, brassinosteroid; CK, cytokinin; JA, jasmonic acid; SA, salicylic acid; GA, gibberellic acid.

Important roles of phytohormones in regulating plant growth and development, and their responses to resist abiotic and biotic stresses have been published by numerous studies [4,20,56,57]. According to the results of MapMan analysis, 108 DEGs including 92 up-regulated and 16 down-regulated genes were shown to be involved in various hormone-related metabolic and signaling pathways (Supplementary Table S7). Interestingly, most hormone-related genes were discovered in the ABA (24), IAA (18), and ethylene (18) metabolic and signaling pathways, followed by those associated with other hormones like GA, JA, CK, brassinosteroid (BR), and salicylic acid (SA) (Supplementary Table S7). As shown in Figure 8, the majority of genes involved in hormone pathways were up-regulated. Particularly, 24 identified genes, including *OvNCED3*, *OvNCED4,* and *OvUGT71B1*, related to ABA pathway all showed up-regulation. Ethylene and IAA were the two other hormones that displayed the second highest number (18 genes/each hormone) of related DEGs, and the majority of these DEG also exhibited up-regulated expression patterns. Our data suggest that the changes in expression of the identified DEGs associated with these hormones, particularly ABA, might contribute to better acclimatization of *O. viciifolia* plants to the adverse environmental conditions of higher altitude at the QTP.

### 3.7. Responses of DEGs Encoding TFs

**Figure 9:**
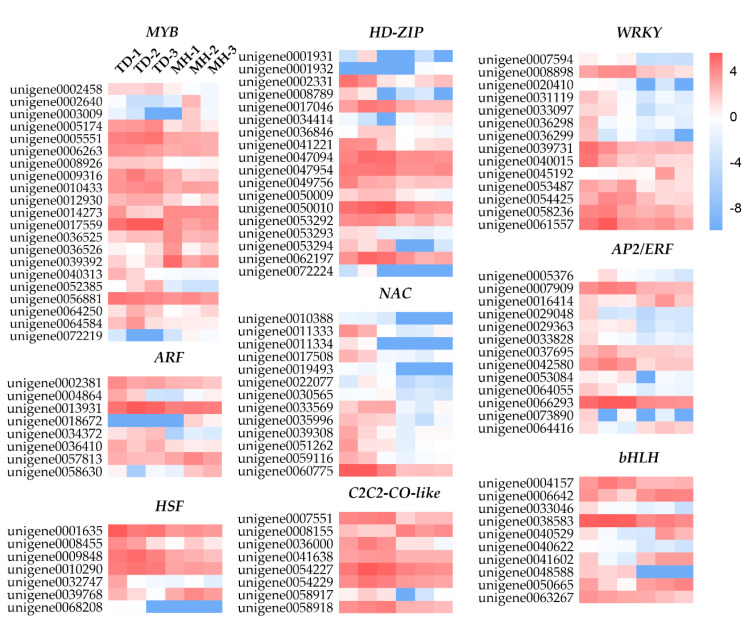
Changes in expression of genes encoding members of several representative transcription factor families in *Onobrychis viciifolia* grown at high-altitude Tongde (TD) and low-altitude Minhe (MH). The heatmaps are constructed according to log_2_ (FC) of differentially expressed genes (TD/MH comparison). Up- and down-regulation are indicated by red and blue, respectively. The colored scale represents expression changes based on the log_2_ (FC) values of TD/MH comparison.

TFs have been shown to play crucial roles in regulating the expression of downstream genes, and impact different physiological characteristics of plants in responses to environmental stresses [20]. In the present study, we identified 155 DEGs putatively referred to as TFs. These TFs were assigned to 21 different TF families, among which 126 TFs were up-regulated and 29 TFs were down-regulated (Supplementary Table S8). The five most abundant TF families were *MYB* (21), *homeodomain-leucine zipper* (*HD-ZIP*, 18), *WRKY* (14), *NAC* (13), and *apetala2/ethylene responsive factor* (*AP2*/*ERF*, 13), followed by *basic helix-loop-helix* (*bHLH*, 10), *C(2)-C(2) zinc finger constans like* (*C2C2*-*CO*-like, 8), *auxin response factor* (*ARF,* 8), *heat shock factor* (*HSF*, 7) and other TF families (43). The majority of *NAC*, *AP2*/*ERF* and *WRKY* family genes in the two group samples presented significantly diverse expression patterns (Figure 9), suggesting that these TFs would functionally play major roles in the environmental stress resistance of *O. viciifolia* grown at high altitude. In comparison, most members of *MYB*, *bHLH*, *C2C2*-*CO-*like, and *HSF* families were highly expressed either in the plants grown at TD or MH. In addition, the majority of *C2C2* (5), *B3* (4), *bZIP* (3), and *gibberellic acid insensitive, repressor of GAI, scarecrow* (*GRAS*, 3) family genes were only highly expressed in the TD-grown plants. In the past decades, the ectopic expression/overexpression of many TFs leading to enhanced resistance to environmental cues in diverse plant species has been reported in numerous studies. For instance, *DREB* genes encoding *AP2/ERF*-type stress-responsive TFs modulate the expression of many downstream stress-inducible genes [58]. Ectopic expression of *StDREB2* of *Solanum tuberosum* in cotton (*Gossypium barbadense*) enhanced the drought resistance, while ectopic expression of *ScDREB10* of *Syntrichia caninervis* in *Arabidopsis* increased osmotic and salt tolerance of transgenic plants. Furthermore, overexpression of *TaDREB3* improved frost resistance of transgenic wheat seedlings [59,60,61]. In addition, *DEAR4* which is a member of the *DREB* subfamily was reported to positively regulate the cell death and leaf senescence, and to be responsive to multiple stressors in *A. thaliana* [62,63]. In our study, unigene0036544, homologous to *DEAR4*, was significantly up-regulated (log_2_ (FC) value of 5.04), which might also be correlated with the responses of *O. viciifolia* to natural conditions in the higher altitude. The TFs identified in our study represent potential targets for promoting *O. viciifolia* plants to be more resistant to the extreme QTP conditions.

### 3.8. Quantitative Analyses of Flavonoids and Anthocyanins

**Figure 10:**
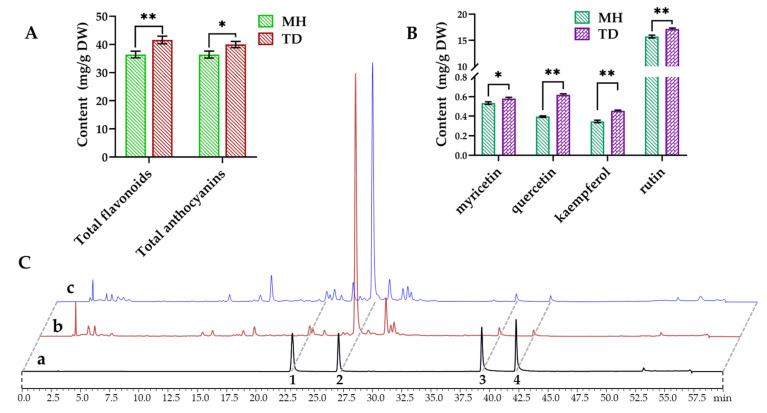
Contents and HPLC profiles of flavonoids and anthocyanins in leaves of *Onobrychis viciifolia* grown at high-altitude Tongde (TD) and low-altitude Minhe (MH). (**A**) Contents of total flavonoids and anthocyanins in TD-grown and MH-grown plant samples. (**B**) Contents of four flavonoids myricetin, quercetin, kaempferol, and rutin. (**C**) HPLC profiles of secondary metabolites (absorption at the wavelength of 275 nm). a, standards; b, TD-grown plants; c, MH-grown plants; 1–4 peaks represent myricetin, rutin, quercetin and kaempferol, respectively. Bars represent means ± SDs of three biological replicates. * and ** indicate statistically significant differences between TD-grown and MH-grown plants at *p* < 0.05 and *p* < 0.01, respectively, as determined by a Student’s *t*-test.

To validate whether the contents of flavonoids and anthocyanins in *O. viciifolia* plants were changed under different altitudes, their total contents were measured using colorimetric method, and four flavonoids including myricetin, quercetin, kaempferol, and rutin were determined by high performance liquid chromatograph (HPLC). Results showed that the contents of total flavonoids and anthocyanins in high-altitude *O. viciifolia* (TD) were 41.58 and 39.96 mg/g dry weight (DW), respectively, which were significantly higher than those (36.75 and 36.42 mg/g DW, respectively) in MH-grown plants at low altitude (Figure 10A and Supplementary Table S9). Furthermore, the contents of four flavonoids also showed higher tendency in the TD- than MH-grown plants. Specifically, myricetin, quercetin, and kaempferol displayed the contents of 0.58, 0.62, and 0.45 mg/g DW, respectively, whereas the respective values were 0.53, 0.4 and 0.35 mg/g DW in the MH-grown plants (Figure 10B,C and Supplementary Table S9). In addition, rutin showed the highest contents among the four compounds with 17.17 and 15.74 mg/g DW in TD and MH, respectively (Figure 10B,C and Supplementary Table S9). These results are agreement with rutin levels previously reported in *O. viciifolia* [32].

## 4. Discussion

As an excellent quality forage legume that has enriched nutritional constituents and anthelmintic properties, and ability to reduce methane emissions and increase nitrogen-use efficiency [28,34], *O. viciifolia* is being increasingly recognized as a high-quality alternative to traditional forage alfalfa in the QTP; and thus, it has a great potential to be widely planted in the QTP. However, the underlying molecular mechanisms of *O. viciifolia* adapting to extreme conditions, especially when growing at higher altitudes, are largely unclear. In this study, using a comparative transcriptome analysis of leaves of *O. viciifolia* plants grown at high altitude (TD) and low altitude (MH), 8,212 DEGs were identified to be involved in the plant responses to higher altitude conditions. This result will greatly increase our understanding with respect to molecular mechanisms underlying acclimatization of *O. viciifolia* plants grown at higher altitudes, enable us to develop adaption-related molecular markers, as well as provide a way to improve quality and yield of this important forage.

The transcriptome of seven-day-old *O. viciifolia* whole seedlings was previously reported, and numerous flavonoid biosynthesis-related genes, simple sequence repeats and single nucleotide polymorphisms were identified [64]. However, the earlier study [64] was conducted using *O. viciifolia* plants grown under the controlled conditions; and thus, the results could not reflect how *O. viciifolia* can respond and adapt to extreme natural conditions in the QTP. Nevertheless, the phylogenetic tree constructed previously showed the closest relationships of *O. viciifolia* with red clover (*Trifolium pratense*) and *M. truncatula* [64]; however, the authors did not include *C. arietinum* in their study. In the present study, *C. arietinum* was included in the phylogenetic analysis, and the results revealed that *O. viciifolia* exhibited the closest relationships to *C. arietinum*, followed by *M. truncatula* and *T. pratense* (Figure 1). Additionally, the annotated sequences in our transcriptome data showed the highest homology to those of *C. arietinum* and *M. truncatula*, further strengthening their closest phylogenetic relationships among others (Table 3).

It is well documented that phenotypic traits of plants are influenced by environmental gradients [65,66,67]. Intriguingly, some phenotypic traits of *O. viciifolia* plants grown at two different altitudes showed a great difference according to our filed survey in the last three years. For example, with the increase of elevation, the plant height and tiller number of *O. viciifolia* plants grown at the high-altitude TD (87.6 cm and 7, respectively) were slightly shorter and much lower, respectively, than those grown at the lower-altitude MH (94.7 cm and 13, respectively) (Supplementary Figure S5). The decrease in plant height along with the altitude elevation was also observed in other species grown in the QTP, including most of the *Rhododendron* species [68], suggesting that this phenotypic change is a common acclimatization strategy of plants growing under adverse natural conditions of the QTP. It was also reported that tiller numbers in two teosintes (*Zea mays ssp. Parviglumis* and *Z. mays ssp. mexicana*) were decreased with the increase of elevation [66]. Since decreased tiller numbers could also be triggered by drought stress [23], drier and cooler climate in the high-altitude TD region might cause decrease in tiller number of *O. viciifolia* plants grown in that area in comparison with those grown in the low-altitude MH. In addition, the flowering time of *O. viciifolia* plants in TD is delayed and the flower longevity is shorter in comparison with MH-grown plants. *O. viciifolia* generally flowers around the beginning of July at the high-altitude TD, whereas it flowers around mid-June at the low-altitude MH. Compared with the MH-grown plants, the flower longevity in the TD-grown plants is shorten by 5 to 10 days based on a filed survey. These observations in phenotypic defects of the TD-grown plants versus the MH-grown plants could be plausibly explained by the cooler weather and longer solar radiation period in higher-altitude regions, which lead to delay in flowering time and shorter flowering period to avoid adverse environmental conditions to allow plants to switch rapidly to the reproductive stage to complete their life cycle [69,70]. Accordingly, transcriptional changes of many circadian rhythm-related genes were detected from the TD/MH comparison. For example, flowering-related genes *OvTOC1* (unigene0002982), *OvFKF1* (unigene0035551), and *OvPRR5* (unigene0001332) were found to be significantly down-regulated with log_2_ (FC) values of −2.12, −2.09, and −1.37, respectively, under long-day photoperiod in the TD/MH comparison (Figure 5). These genes are believed to negatively regulate flowering time in *A. thaliana* and rice (*Oryza sativa*) [23,71,72,73,74], suggesting that down-regulation of these genes may lead to delaying flowering time in *O. viciifolia* plants grown at the high-altitude TD as discussed earlier. Furthermore, *OvPIF3* and its target gene *OvLHY* also negatively regulate flowering time in *Arabidopsis* [75]. Interestingly, unigene0042002 and unigene0067673 homologous to *OvPIF3* and *OvLYH*, respectively, were up-regulated in TD/MH comparison in this study (Figure 5), implying a complex circadian rhythm-related mechanism regulating late flowering of *O. viciifolia* plants growing at high altitudes with extreme natural conditions.

**Figure 11:**
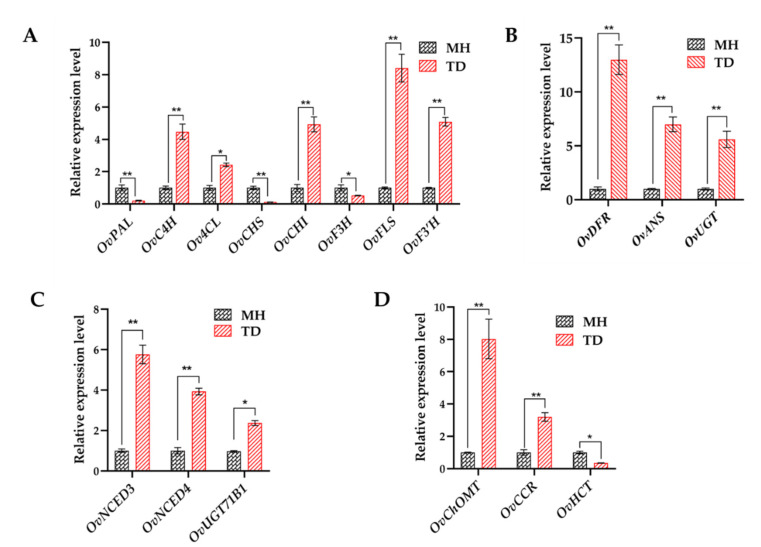
Relative expression levels of key genes involved in the metabolism of flavonoids, anthocyanins, phenylpropanoid and ABA in leaves of *Onobrychis viciifolia* under high-altitude Tongde (TD) and low-altitude Minhe (MH). (**A**) Flavonoid metabolism-related genes. (**B**) Anthocyanin metabolism-related genes. (**C**) Phenylpropanoid metabolism-related genes. (**D**) ABA metabolism-related genes. The expression level of each gene in MH-grown plants was set as 1. Bars represent means ± SDs of three biological replicates. * and ** indicate statistically significant differences between TD-grown and MH-grown plants at *p* < 0.05 and *p* < 0.01, respectively, as determined by a Student’s *t*-test.

Plant secondary metabolites, in particular flavonoids, phenylpropanoids, and anthocyanins that all derive from phenylalanine biosynthetic pathway, play essential roles in resisting environmental stimuli [76,77]. Moreover, flavonoids and anthocyanins have critical functions in regulating fruit colors and qualities. MapMan analysis of 8212 DEGs displayed that flavonoid, anthocyanin, and phenylpropanoid biosynthetic pathways were the most highly enriched with the DEGs among all detected secondary metabolite pathways, and the majority of related genes were up-regulated (Supplementary Table S7), suggesting the importance of these three classes of polyphenols in acclimatization of *O. viciifolia* to high-altitude conditions. Generally, when facing with detrimental environmental stresses, plants initiate a wide range of enzymatic and non-enzymatic antioxidants to scavenge excess free radicals, including ROS [54,78,79]. The polyphenolic compounds are the central components of non-enzymatic antioxidants [54]; and thus, promotion of their biosynthesis is a defense strategy against adverse environmental conditions [76,80,81]. Consistent with the results of transcriptome analysis, results of RT-qPCR analysis also verified up-regulation of several key flavonoid, phenylpropanoid, and anthocyanin biosyntheses-related genes, especially *OvFLS*, in the TD/MH comparison (Figure 11A,B).

Intriguingly, although *OvPAL*, *OvCHS,* and *OvF3H*, which are involved in flavonoid biosynthesis, showed decreased expression levels in TD/MH comparison (Figure 11A), the content of total flavonoids in TD-grown plants was significantly higher than in MH-grown plants, indicating a complex regulation of flavonoid biosynthesis where these three genes might not play rate-limiting roles. Nevertheless, this result demonstrates the importance of enhanced flavonoid biosynthesis in acclimatization of *O. viciifolia* to high-altitude conditions. In addition, three end-products of OvFLS enzyme, namely myricetin, quercetin, and kaempferol, were also highly produced in the TD- than MH-grown plants (Figure 10 and Supplementary Table S9). This result suggests that low molecular weight antioxidants are more highly produced to help *O. viciifolia* resist oxidative stress triggered by extreme natural conditions at higher altitudes. In addition to the three above-mentioned flavonoids, the compound rutin, a glycosylated derivative of quercetin, displayed the highest contents in both TD- and MH-grown plants, which TD-grown plants possessing higher rutin content than MH-grown plants (Figure 10 and Supplementary Table S9). A recent investigation also revealed the critical roles of rutin in abiotic stress tolerance of *Fagopyrum tataricum* [82]. It is plausible that induction of rutin biosynthesis in *O. viciifolia* is important for its adaption to extreme natural conditions at high altitudes in the QTP as well.

In comparison with MH, three key genes, namely *OvDFR*, *OvANS,* and *OvUGT*, that are involved in anthocyanin biosynthesis were up-regulated in the range of 5- to 13-fold in leaves of TD-grown versus MH-grown *O. viciifolia* plants (Figure 11B), which is consistent with the observed higher anthocyanin contents in TD plants than MH plants (Figure 10A). In the phenylpropanoid biosynthetic pathway, the *OvChOMT* and *OvCCR* were also dramatically up-regulated, whereas *OvHCT* was down-regulated in the TD/MH comparison (Figure 11C), which might be explained by the fact that the biosynthesis of phenylpropanoid is independent of HCT enzymes [83]. Taken together, our results indicate that, the biosyntheses of polyphenolic antioxidants are activated in *O. viciifolia* along with increasing altitudes to adapt to more extreme natural conditions.

Important roles of phytohormones in regulating plant growth and development, and their responses to resist abiotic and biotic stresses have been published by numerous studies [4,20,56,57]. Notably, in the ABA biosynthetic pathway, two genes *OvNCED3* (unigene0064567) and *OvNCED4* (unigene0008736) belonging to the *nine-cis-epoxycarotenoid dioxygenase* (*NCED*) family, were both significantly up-regulated in TD/MH comparison with log_2_ (FC) values of 3.40 and 3.10, respectively (Supplementary Table S7), as also verified by RT-qPCR (Figure 11D). Members of the *NCED* family have been shown to encode key rate-limiting enzymes through the oxidative cleavage of *cis*-epoxycarotenoids in the ABA biosynthetic pathway to accumulate ABA for further signal transduction in order to resist abiotic stresses [84,85,86,87]. In recent studies, overexpression of *AtNCED3* in *Arabidopsis* enhanced the drought tolerance by promoting the levels of ABA [88,89]. In rice, the overexpression of *OsNCED3* resulted in increased ABA content and promote water stress tolerance of the transgenic plants [90]. These results clearly demonstrated a positive correlation between expression levels of *NCED* genes and ABA accumulation, which suggests that up-regulation of *OvNCED3* and *OvNCED4* in TD/MH comparison might accelerate ABA biosynthesis and accumulation in TD-grown *O. viciifolia* plants, compared with MH-grown plants, leading to their enhanced resistance to adverse climate conditions at high altitude of the QTP. In addition, the majority of the IAA-related genes identified showed homology to *small auxin-up RNA* (*SAUR*) genes, among which unigene0034372 showing high homology to *SAUR31* of *A. thaliana* was the most highly up-regulated gene (log_2_ (FC) = 5.02) (Supplementary Table S7). Auxin affects almost every aspect of plant development, and is also an important regulator of the interaction between plants and their growth environment [91]. During the regulation processes by auxin, *SAUR* genes are of paramount importance in the regulation of dynamic and adaptive growth in plant responses to environmental cues [92]. Our data suggested that the highly induced expression levels of putative *SAUR* genes in *O. viciifolia* might also be linked to its responses to the extreme environmental conditions of the QTP.

Uridine diphosphate glucosyltransferases (UGTs) are extensively believed to modulate the endogenous phytohormone homeostasis to further promote abiotic stress resistance in plants via their glycosylated function that is referred to the formation of hormone conjugates [93,94,95,96,97,98]. As expected, numerous DEGs encoding UGTs were identified to participate in various hormone-related pathways (Figure 8 and Supplementary Table S7). For instance, according to the transcriptome data, unigene0037761, encoding OvUGT71B1 involved in ABA metabolic pathway, was up-regulated (log_2_ (FC) = 2.96) in the TD/MH comparison (Supplementary Table S7), as also confirmed by RT-qPCR analysis (Figure 11D). A previous study reported that three *UGT* genes of *A. thaliana*, namely *UGT71B6*, *UGT71B7,* and *UGT71B8*, to which *OvUGT71B1* shows high homology, play pivotal roles in regulating ABA homeostasis, and in *Arabidopsis* adaptation to osmotic stress, dehydration and salinity [94,96]. In the CK-related pathway, two OvUGT-encoding genes, unigene0018949 and unigene0055908 coding for OvUGT85A2 and OvUGT73C1, were up-regulated with log_2_ (FC) values of 2.60 and 1.60, respectively, while unigene0039749 encoding OvUGT85A1 was significantly down-regulated with the log_2_ (FC) value of −8.92 (Figure 8 and Supplementary Table S7). Additionally, all eight SA metabolism-related DEGs were up-regulated with the log_2_ (FC) values in the range of 1.09 to 1.81 (Figure 8 and Supplementary Table S7). Six of these DEGs putatively encode OvUGT74F1 that produces the SA conjugate in the form of SA 2-*O*-β-D-glucose [99]. Taken together, these results suggest that the identified putative *OvUGT*s, including *OvUGT71B1*, *OvUGT85A2*, *OvUGT73C1*, *OvUGT85A1,* and *OvUGT74F1* (Supplementary Table S7), might act to adjust the levels of different hormones in *O. viciifolia*, thereby contributing to its proper growth and resistance to the adverse natural conditions of the high altitude of QTP. Such adjustment in the expression levels of genes related to the hormone dynamic detected in the *O. viciifolia* plants grown at the high altitude versus low altitude was probably due to continuous climate fluctuation at the higher altitude.

## 5. Conclusions

To survive, plants have evolved different machineries to respond and adapt to the ever-changing environments. An in-depth understanding of the molecular mechanisms involved in plant responses to diverse environmental stresses will enable us to improve plant stress resistance via genetic engineering, and subsequently promote the quality and productivity of crops. In our study, a total of 8212 DEGs, including 5387 up- and 2825 down-regulated genes, were identified from leaves of *O. viciifolia* grown at a higher altitude (TD) in comparison to a lower altitude (MH) in the QTP for the first time using the RNA-seq technology. These DEGs were revealed to be involved in various stress response-related processes, including hormone metabolism, circadian rhythm, secondary metabolite metabolism, and signaling transduction pathways. Particularly, numerous identified genes were found to be participated in the flavonoid and phenylpropanoid biosynthesis, suggesting the involvement of these compounds in *O. viciifolia* responses to the more extreme natural conditions at higher altitude of the QTP. Furthermore, many genes related to hormone metabolism and signaling, as well as regulatory networks modulated by different types of TFs were found to participate in *O. viciifolia* responses to adverse natural conditions at higher altitudes. In conclusion, the transcriptome datasets obtained from the leaves of *O. viciifolia* plants field-grown at two different altitudes of the QTP allowed us to identify a series of candidate genes associated with their differential responses to environmental stresses under natural conditions, which could provide the basis for understanding and further elucidating the molecular regulatory mechanisms involved in *O. viciifolia* acclimatization to the QTP extreme environment.

## Figures and Tables

**Table 1:** Summary of the de novo assembly of transcriptome sequence reads from the TD- and MH-grown plants. TD and MH indicated high-altitude and low-altitude samples collected from the Tongde and Minhe regions located in the Qinghai-Tibetan Plateau, respectively. Q20 and Q30 are referred to the base call accuracy rate of 99% and 99.9%, respectively. N50 is defined as the read length when such reads added to all the longer reads reach to 50% sum of reads of all lengths.

Content	Total	TD-1	TD-2	TD-3	MH-1	MH-2	MH-3
**Total raw reads**	57,014,250	44,392,764	81,922,056	69,023,986	53,001,304	44,778,542	48,966,846
**Total clean reads**	55,645,070	43,177,362	79,839,438	67,258,662	51,867,538	43,809,044	47,918,378
**GC percentage (%)**		43.25%	43.43%	44.07%	44.08%	43.67%	43.94%
**Clean reads Q20 (%)**		97.51%	97.66%	97.66%	97.76%	97.76%	97.80%
**Clean reads Q30 (%)**		93.59%	93.95%	93.95%	94.09%	94.14%	94.22%
**Clean reads (%)**		97.26%	97.46%	97.44%	97.86%	97.83%	97.86%
**Total mapped reads (%)**		88.04%	88.71%	89.16%	89.54%	88.91%	89.33%
**Total expressed genes**	74,791	64,331	62,887	59,219	56,903	60,218	60,218
**Total transcripts**	218,482						
**Total unigenes**	74,791						
**N50 (bp)**	1458						
**Mean length of unigenes (bp)**	820						

**Table 2:** Number of unigenes annotated using the public databases.

Annotated Database	Number of Unigenes	Percentage (%)
**Annotated in Nr**	43,717	58.45
**Annotated in SwissPort**	32,973	44.09
**Annotated in KOG**	24,841	33.21
**Annotated in KEGG**	16,305	21.80
**Annotated in all databases**	46,508	62.18
**All assembled unigenes**	74,791	100

**Table 3:** Summary of annotations of assembled *Onobrychis viciifolia* consensus sequences.

Species	Number of Matched Unigenes	Percentage
***Medicago truncatula***	11,592	24.92%
***Cicer arietinum***	9758	20.98%
***Glycine max***	3553	7.64%
***Cajanus cajan***	3358	7.22%
***Glycine soja***	1733	3.73%
***Vigna angularis***	1113	3.39%
***Vigna radiata***	1109	2.38%

## Supplementary Materials

The following are available online at https://www.mdpi.com/2218-273X/10/6/967/s1, Figure S1: Length distribution of assembled unigenes, Figure S2: Venn diagram of functional annotations of unigenes using various public databases, Figure S3: Relative expression levels of selected genes used for validating the accuracy of RNA-seq data obtained from leaves of *Onobrychis viciifolia* under high (Tongde, TD) and low altitudes (Minhe, MH). Figure S4: Key differentially expressed genes involved in the phenylpropanoid pathway, Figure S5: Comparison of height and tiller number of *Onobrychis viciifolia* plants grown under high (Tongde, TD) and low altitudes (Minhe, MH), Table S1: Information on the 5387 up- and 2825 down-regulated unigenes in leaves of *Onobrychis viciifolia* plants grown at high-altitude Tongde (TD) vs. those grown at low-altitude Minhe (MH), Table S2: Top 50 DEGs obtained from the comparison of *Onobrychis viciifolia* plants grown at high-altitude Tongde (TD) and low-altitude Minhe (MH), Table S3: GO enrichment analysis of differentially expressed genes, Table S4: Enriched KEGG pathways of differentially expressed genes, Table S5: Metabolic pathway enrichment analysis of differentially expressed genes identified in leaves of *Onobrychis viciifolia* grown at high-altitude Tongde (TD) vs. those grown at low-altitude Minhe (MH), Table S6: Mapman annotation of key differentially expressed genes involved in flavonoid and phenylpropanoid biosynthetic pathways, Table S7: Differentially expressed genes involved in hormone pathways as annotated by MapMan analysis, Table S8: Differentially expressed genes encoding transcriptional factors identified from transcriptome analysis of *Onobrychis viciifolia* leaf samples, Table S9: Contents of total flavonoids, anthocyanins, and other flavonoid compounds found in leaves of *Onobrychis viciifolia* plants grown at high-altitude Tongde (TD) and low-altitude Minhe (MH), Table S10: Unigenes and their specific primers used in RT-qPCR analysis.
