# Supplementary material for: Transcriptome Analysis Reveals Potential Roles of Abscisic Acid and Polyphenols in Adaptation of Onobrychis viciifolia to Extreme Environmental Conditions in the Qinghai-Tibetan Plateau
Source: Biomolecules. 2020 Jun 26;10(6):967. doi: 10.3390/biom10060967 (PMC7356597; doi:10.3390/biom10060967)
Supplement: Supplementary file 1 [file biomolecules-10-00967-s001.zip › Supplementary data_R1_0622/Supplementary Figures_R1_0622.docx]

**Supplementary Figures**


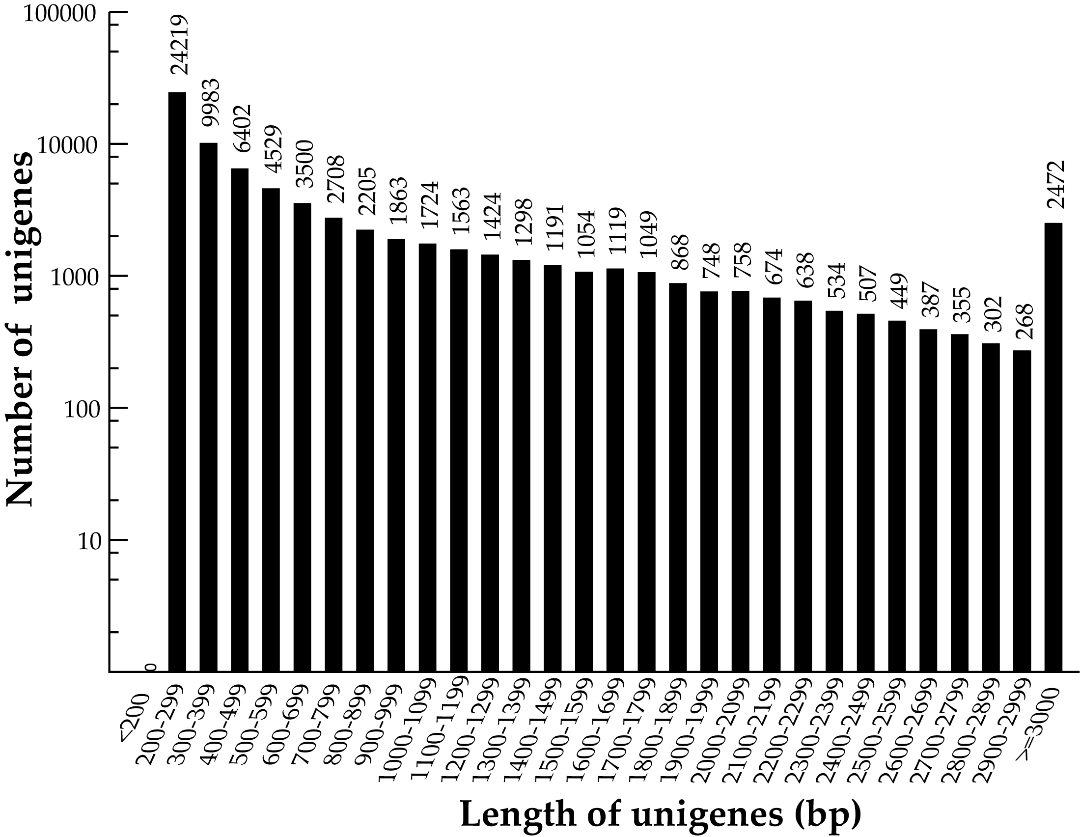


**Figure S1.** Length distribution of assembled unigenes. The numbers above histograms indicate the numbers of genes distributed in different ranges of gene length. X-axis indicates the ranges of assembled unigene length. Y-axis indicates the number of corresponding unigenes.


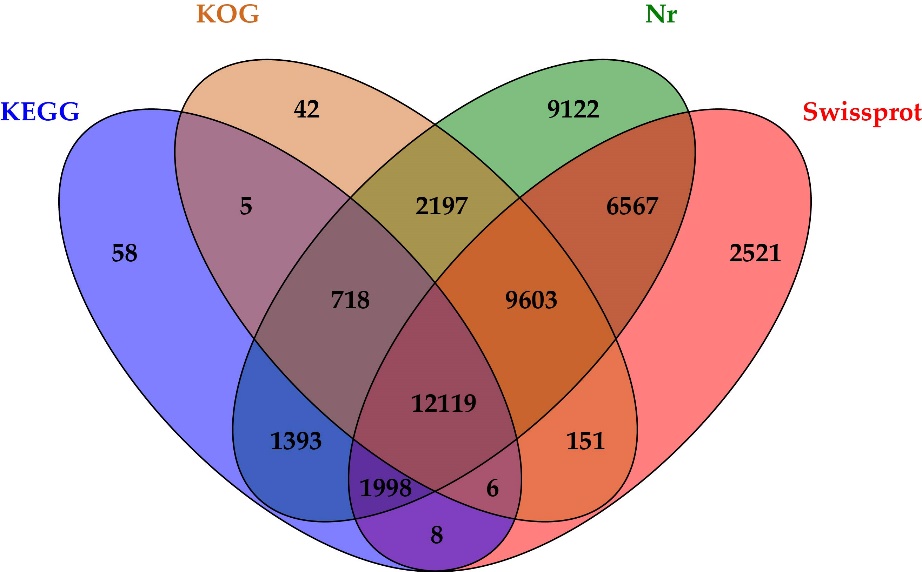


**Figure S2.** Venn diagram of functional annotations of unigenes using various public databases. KEGG, Kyoto Encyclopedia of Genes and Genomes; KOG, EuKaryotic Orthologous Groups; Nr, Non-Redundant Protein Sequence Database; SwissProt, Swiss Institute of Bioinformatics databases. The sum of numbers in each oval represents total genes annotated by corresponding database. The numbers within cross ovals indicate genes co-annotated by different databases.


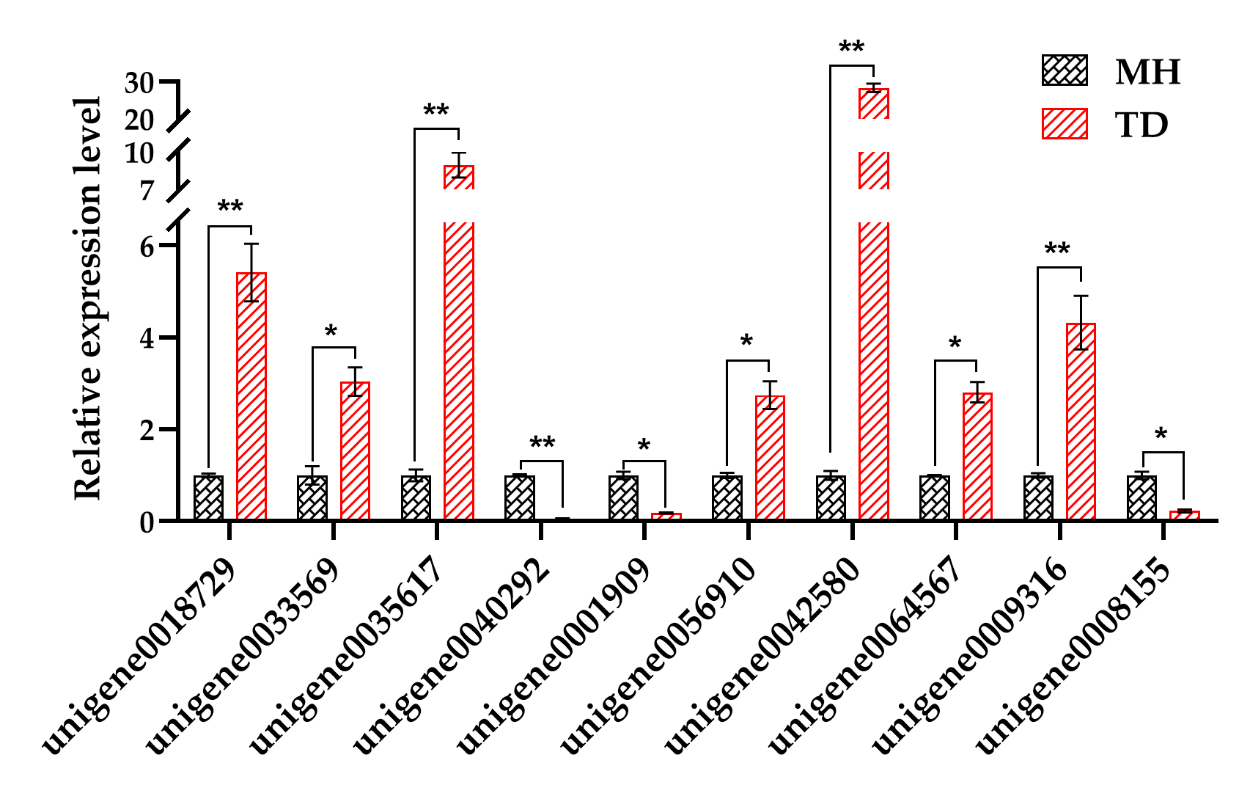


**Figure S3.** Relative expression levels of selected genes used for validating the accuracy of RNA-seq data obtained from leaves of *Onobrychis viciifolia* under high (Tongde, TD) and low altitudes (Minhe, MH). The expression level of each gene in MH-grown plants was set as 1. Bars represent means ± SDs of three biological replicates. * and ** indicate statistically significant difference between TD-grown and MH-grown plants at *p* < 0.05 and *p* < 0.01, respectively, as determined by a Student’s *t*-test.


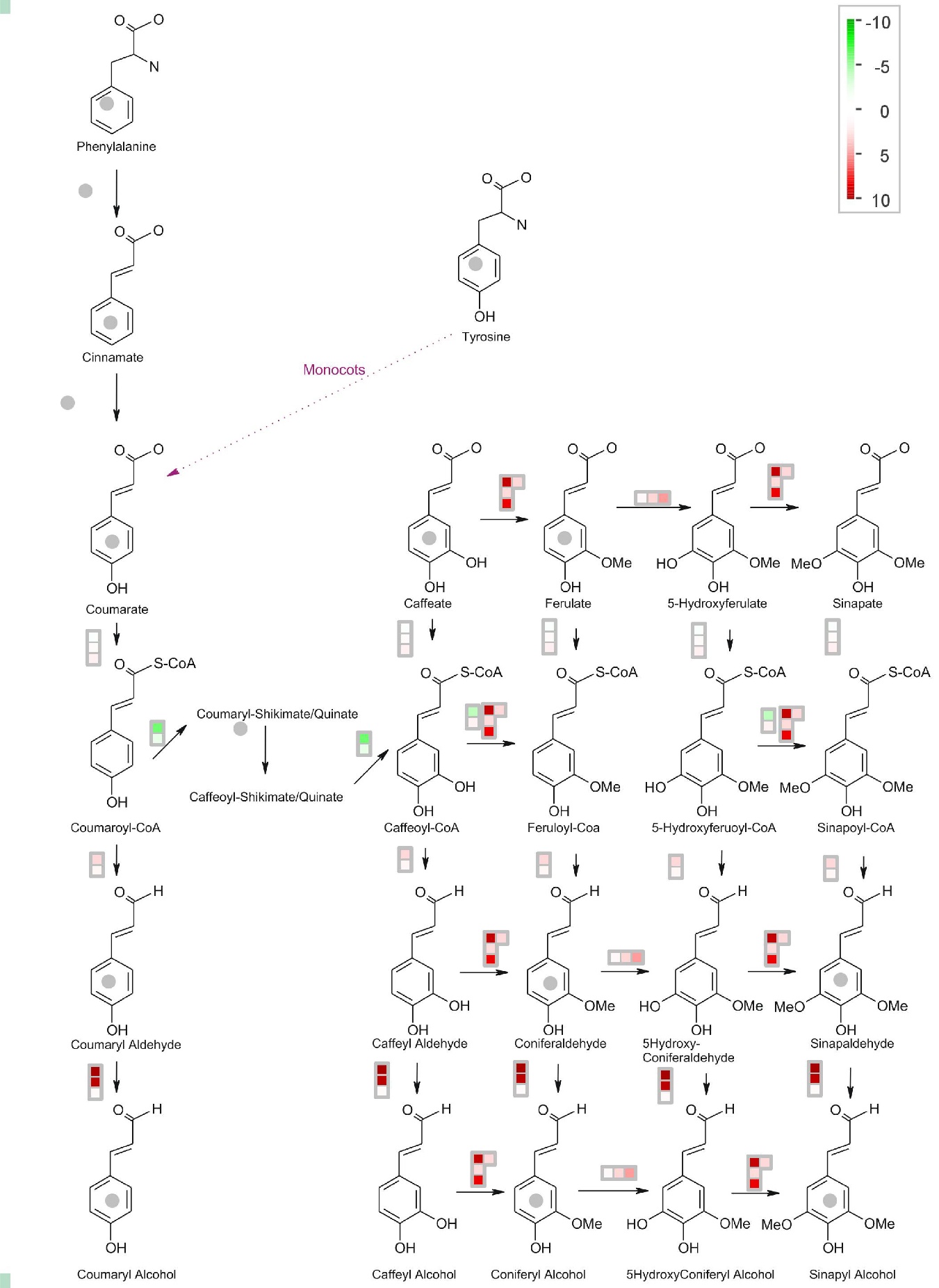


**Figure S4.** Key differentially expressed genes involved in the phenylpropanoid pathway. Colored boxes and scale indicate expression changes of related genes in Tongde (TD)-grown plants compared with Minhe (MH)-grown plants. Red and green colors represent up- and down-regulation, respectively, based on log_2_ (FC) in expression values of related genes (TD/MH comparison).


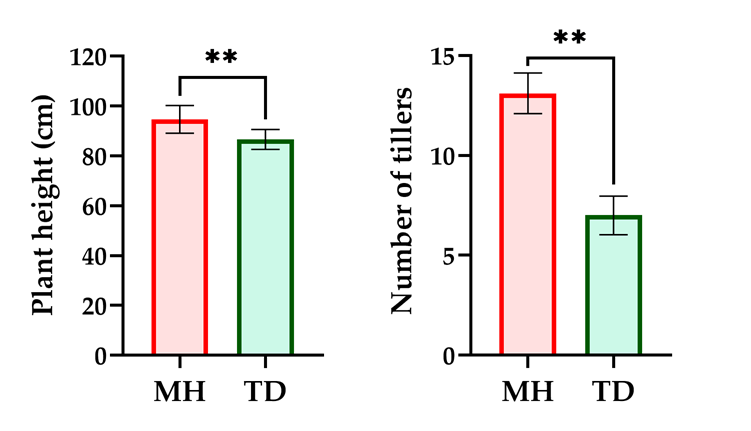


**Figure S5.** Comparison of height and tiller number of *Onobrychis viciifolia* plants grown under high (Tongde, TD) and low altitudes (Minhe, MH). Data are means ± SDs. For each location, n > 10 in three independent sampling areas. ** indicate statistically significant differences between TD-grown and MH-grown plants at *p* < 0.01, as determined by a Student’s *t*-test.
